# Retraction: Involvement of Hus1 in the chain elongation step of DNA replication after exposure to camptothecin or ionizing radiation

**DOI:** 10.1093/nar/gkaa119

**Published:** 2020-02-25

**Authors:** Xiang Wang, Jun Guan, Baocheng Hu, Robert S Weiss, George Iliakis, Ya Wang

**Affiliations:** 1 Department of Radiation Oncology, Kimmel Cancer Center of Jefferson Medical College, Thomas Jefferson University, Philadelphia, PA 19107, USA; 2 Department of Biomedical Sciences, Cornell University, Ithaca, NY 14853, USA; 3 Institute of Medical Radiation Biology, University of Duisburg-Essen Medical School, 45122 Essen, Germany

*Nucleic Acids Research* (2004) 32(2): 767–775, doi: https://doi.org/10.1093/nar/gkh243

In Figure 4B, multiple bands have been duplicated. While these irregularities may not fundamentally affect the results and conclusions, the Editors nonetheless note that such manipulation of data and corresponding figures is unacceptable, and request retraction of the article.

